# Establishing super-resolution imaging for proteins in diatom biosilica

**DOI:** 10.1038/srep36824

**Published:** 2016-11-09

**Authors:** Philip Gröger, Nicole Poulsen, Jennifer Klemm, Nils Kröger, Michael Schlierf

**Affiliations:** 1 B CUBE–Center for Molecular Bioengineering, TU Dresden, Arnoldstr. 18, 01307 Dresden, Germany; 2 Department of Chemistry and Food Chemistry, TU Dresden, Arnoldstr. 18, 01307 Dresden, Germany

## Abstract

The intricate, genetically controlled biosilica nano- and micropatterns produced by diatoms are a testimony for biology’s ability to control mineral formation (biomineralization) at the nanoscale and regarded as paradigm for nanotechnology. Previously, several protein families involved in diatom biosilica formation have been identified, and many of them remain tightly associated with the final biosilica structure. Determining the locations of biosilica-associated proteins with high precision is, therefore expected to provide clues to their roles in biosilica morphogenesis. To achieve this, we introduce here single-molecule localization microscopy to diatoms based on photo-activated light microscopy (PALM) to overcome the diffraction limit. We identified six photo-convertible fluorescent proteins (FPs) that can be utilized for PALM in the cytoplasm of model diatom *Thalassiosira pseudonana*. However, only three FPs were also functional when embedded in diatom biosilica. These were employed for PALM-based localization of the diatom biosilica-associated protein Silaffin-3 (tpSil3) with a mean precision of 25 nm. This allowed for the identification of distinct accumulation areas of Sil3 in the biosilica, which cannot be resolved by confocal fluorescence microscopy. The enhanced microscopy technique introduced here for diatoms will aid in elucidating the molecular mechanism of silica biomineralization as well as other aspects of diatom cell biology.

Diatoms are one of the most abundant unicellular microalgae found in all aquatic ecosystems across the world^1^. A distinct feature of diatoms is their silica-based cell wall that exhibits an intricate multi-scale pattern with structural elements spanning from micrometers down to tens of nanometers. The morphogenesis of the diatom biosilica is a highly complex process that occurs with the help of self-assembled biosilica forming templates^2^. Over the past decade, biochemical studies have identified several organic components (i.e. silaffins, cingulins, silacidins, SiMat proteins, long-chain polyamines, chitin) that are tightly associated with the diatom silica and believed to be involved in formation of the species-specific silica nano- and micropatterns^3^,^4^,^5^,^6^,^7^,^8^. Live cell imaging of the peptides and proteins thought to be involved in cell wall formation has become possible over the past years due to the development of methods for their genetic transformation^9^. Through the expression of GFP fusion proteins in the model diatom *Thalassiosira pseudonana*, cell wall associated proteins have been localized to distinct regions of the biosilica. For example, the silaffin tpSil3 is associated with the so called valve region whereas the cingulins are associated with the girdle band region (Fig. 1)^7^. However, these studies have not been able to determine the precise localization of the GFP-fusion proteins as confocal imaging is limited by the diffraction limit of light (≈250 nm) (Fig. 1B). To visualize the intricate nanometer-sized silica pattern, typically electron microscopy is used^10^,^11^,^12^. While electron microscopy offers unparalleled spatial resolution, it is very challenging to use this imaging technique for the visualization of silica embedded proteins and impossible to perform imaging with live cells (Fig. 1A). Fluorescence microscopy, on the other hand, allows live cell imaging and direct access to simultaneous multi-color and thus multi-protein visualization. Recently, structured illumination microscopy with its resolution of up to 150 nm has been applied to localize fusion proteins inside diatoms^13^. Yet, single-molecule localization microscopy (SMLM) which extends the range of fluorescence microscopy to spatial resolutions in the tens of nanometer regime^14^,^15^, has not been applied to diatoms. Both photo-activatable localization microscopy (PALM) and stochastic optical reconstruction microscopy (STORM) employ temporal and spatial separation of single-fluorophores to reconstruct a high resolution representation from a series of hundreds of individual images^16^. While STORM is based on immunofluorescence localization of proteins using antibodies, PALM offers the opportunity to use fusion proteins based on switchable fluorescent proteins, with the benefit of an intrinsic high labeling density. SMLM is able to bridge the resolution gap between confocal and electron microscopy. Therefore, super-resolution microscopy is particularly interesting for studying diatom biomineralization, since the feature sizes of biosilica range typically between ten and several hundred nanometers.

Here, we have established SMLM for the diatom *T. pseudonana* with the aim of visualizing the precise localizations of protein-based templates inside the biosilica. As the silica may limit antibody access to the protein of interest, we chose to use PALM, which is based on the expression of fusion proteins with photo-convertible fluorescent proteins. Over the past years, a large variety of switchable fluorescent proteins have been developed^17^,^18^. We have screened six different fluorescent proteins (FPs) to identify and evaluate suitable candidates. To this end, we compared the photo-conversion abilities of cytosolic FPs with that of biosilica-embedded FPs. For the biosilica embedding we created fusion proteins with Silaffin-3 (tpSil3), which is thought to be involved in biosilica formation and remains permanently entrapped inside the biosilica of the valve region^19^,^20^. Unexpectedly, we have found that only a subset of the photo-convertible proteins can be used when embedded in the biosilica. In contrast, all FPs could be activated efficiently in the cytosol. Reconstruction microscopy on the single-molecule level allowed for localization of Dendra2, mEOS3.2 and Dronpa fusion-proteins embedded in the silica with an average precision of 28 nm, 25 nm and 25 nm, respectively.

## Results

### Photo-conversion of fluorescent proteins in the cytoplasm of living diatoms

To find suitable fluorescent proteins (FPs) as super-resolution probes for biosilica embedded fusion-proteins, we chose to screen six different candidates, namely PATagRFP^21^, PAmCherry1^22^, PA-GFP^23^, mEOS3.2^24^, Dendra2^25^ and Dronpa^26^ (Table 1 and SI Table S1). PATagRFP, PAmCherry1 and PA-GFP are photo-activatable FPs that undergo an activation from a non-fluorescent state to a fluorescent state, whereas mEOS3.2 and Dendra2 are photo-convertible FPs that can be converted from one fluorescent to another fluorescent state with a red-shifted excitation and emission spectrum. Dronpa is a photo-switchable FP that can be repeatedly switched between a non-fluorescent and a fluorescent state.

In particular, we focused on a range of different emission wavelengths, since diatom chloroplasts exhibit a broad autofluorescent background that could interfere with imaging of the FPs. The chloroplast absorbance of *T. pseudonana* peaks at λ_exc_ ≈ 450 nm (chlorophyll c and fucoxanthin) and at λ_exc_ ≈ 670 nm (chlorophyll a) with an emission ranging roughly from 650 nm to 750 nm (SI Fig. S1) preventing imaging of FPs emitting in those wavelength windows. Another important selection criterion was a strict monomericity of the FPs, to avoid unnatural aggregation of the silaffin fusion proteins during silica formation. Thus, we excluded previously reported multimeric FPs like Kaede^27^, KFP1^28^, or IrisFP^29^.

In a first set of experiments, we tested the photo-activation of all candidate proteins expressed in the cytosol of diatoms. We inserted the FP of choice into the expression vector pTpfcp^30^ for constitutive cytosolic expression in *T. pseudonana* (for a scheme of all vectors used for cloning see SI Fig. S7). Successful transformants (at least 25% of the screened clones showed fluorescence) were identified by fluorescence microscopy and further tested for an activatable fluorescence signal in the cytosol after brief illumination with λ_act_ = 405 nm. All six FPs (PATagRFP, PAmCherry1, PA-GFP, mEOS3.2, Dendra2 and Dronpa) could be activated, converted or switched to their second state (Table 1 and SI Fig. S2). As examples, the successful activation of PA-GFP from an initial dark state to its green state, conversion of mEOS3.2 from the initial green state to the orange state and switching of Dronpa from the dark to the green state after a 1 s illumination at λ_act_ = 405 nm are shown in Fig. 2A-C (see SI Fig. S2 for an overview of all FPs used in this study).

### Photo-conversion of biosilica-embedded proteins in live diatoms

In a second set of experiments, we tested the activation of the FPs when embedded in the diatom biosilica. For that purpose, recombinant genes encoding C-terminal fusion proteins of the FPs with tpSil3 were constructed, stably incorporated into the *T. pseudonana* genome, and expressed under control of the constitutive fcp promoter (see SI Fig. S7). When tpSil3 is expressed under the control of the fcp regulatory elements, it is incorporated into all regions of the biosilica^31^. In contrast to cytosolic FP expression, only three of the six FPs that were tested were convertible when fused to tpSil3 (Table 1, SI Figs S2 and S3). In particular, the three FPs that require photo-activation from a dark state (i.e. PATagRFP, PAmCherry1 and PA-GFP) were not activatable–even after multiple seconds of λ_act_ = 405 nm exposure (see Fig. 2C showing PA-GFP as an example). By screening at least 24 clones and multiple cells per clone, it is very likely that the absence of fluorescence originates from a hindered photo-activation rather than a failed genetic transformation.

In contrast, all the FPs that are either photo-convertible or photo-switchable (i.e. mEOS3.2, Dendra2, Dronpa) were successfully converted from the green emission state or dark state to the orange emission state. Examples of a successful photo-conversion of mEOS3.2 and photo-switching of Dronpa are shown in Fig. 2E,F, respectively, where tpSil3-mEOS3.2 was converted and tpSil3-Dronpa was switched with a 1 sec activation with λ_act_ = 405 nm. As in the cytosolic construct, the conversion happens during a single frame of UV illumination and seems not to be hindered by the silica-embedment. A time course analysis of photo-conversion over multiple cycles of UV illumination showed a rapid saturation of converted fluorescence within a few cycles for all three fusion proteins (SI Fig. S3).

The observed difference in photo-convertibility might originate from the underlying molecular mechanisms. Indeed, the conversion mechanism of PA-GFP, PATagRFP and PAmCherry1 relies on a decarboxylation of Glu222^32^,^33^,^34^. This glutamic acid sits in the β-barrel of the protein and is most likely partly exposed to the silica environment. Thus, it is conceivable that the surrounding silica environment impairs the decarboxylation. Interestingly, the three FPs that were converted inside the biosilica do not require this decarboxylation–they rely on a breakage of the polypeptide backbone near the chromophore (mEOS3.2, Dendra2)^35^ or a cis-trans isomerization of the chromophore (Dronpa)^36^. These three conversions occur in the center of the β-barrel, which is most likely protected from the surrounding biosilica.

### Super-resolution microcopy of silica embedded fusion proteins

**Figure 1:**
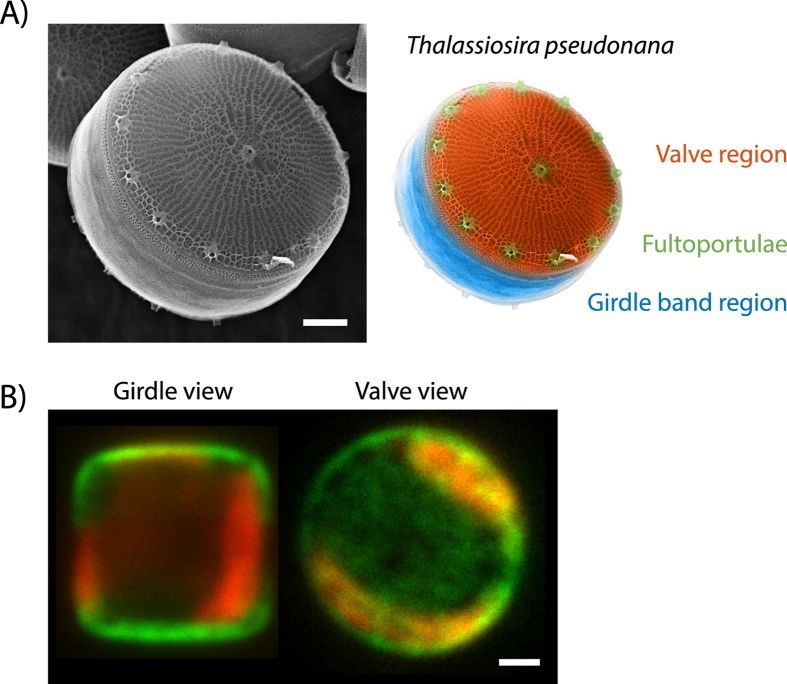
The diatom *T. pseudonana.* (**A**) Left: SEM image of the *T. pseudonana* biosilica displaying parts of the girdle band region and the planar valve region featuring the prominent fultoportulae and a ridge network. Right: False coloring of the EM picture to highlight structural features of the *T. pseudonana* biosilica. (**B**) *In vivo* confocal microscopy images of two *T. pseudonana* cells with the GFP-tagged Sil3 protein in green (488 nm excitation) in ‘girdle view’ (left) and ‘valve view’ (right) orientation. The red fluorescence is caused by autofluorescence of the chloroplasts (647 nm excitation). All scale bars are 1 μm.

**Figure 2:**
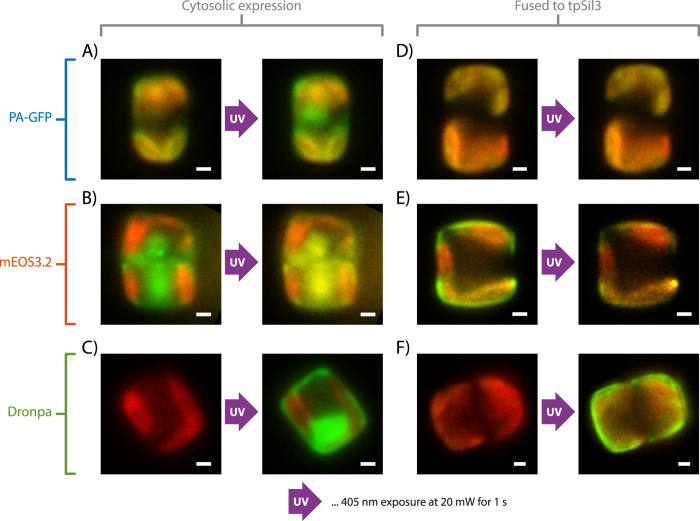
Epifluorescence images of photo-conversion. The red channel (647 nm excitation) always shows the chloroplast emission. (**A**) Photo-activation of cytosolic PA-GFP. The Green channel (488 nm excitation) shows activated PA-GFP after UV exposure. (**B**) Photo-conversion of cytosolic mEOS3.2. The Green channel shows unconverted mEOS3.2, which is converted via λ_act_ ≈ 405 nm to its red shifted form that can be excited at 561 nm (yellow channel). (**C**) Photo-switching of cytosolic Dronpa. The green channel shows switching of Dronpa between its dark and active state. (**D**) TpSil3-PA-GFP is embedded in biosilica and cannot be activated with UV light. (**E**) TpSil3-mEOS3.2 embedded in biosilica (green). Embedded tpSil3-mEOS3.2 can be efficiently converted (yellow). (**F**) TpSil3-Dronpa can be converted. All scale bars are 1 μm.

**Figure 3:**
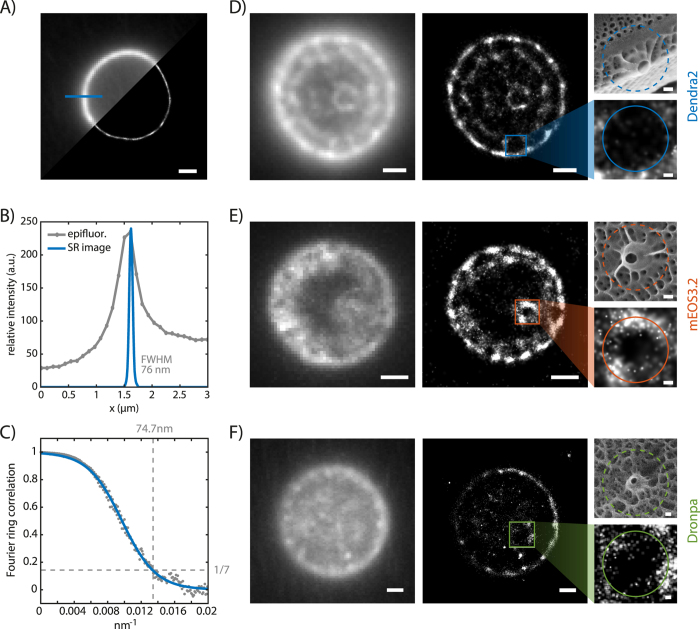
PALM on tpSil3. (**A**) Comparison of an epifluorescence image and the reconstructed super-resolution image of tpSil3-Dendra2 with z-focus on the girdle band area of the diatom. The position of the line scan is highlighted. (**B**) Line scan through the silica cell wall showing the fluorescence intensity profile for both imaging modalities. The FWHM of the PALM image is denoted. (**C**) Fourier ring correlation for the super-resolution image shown in A reveals an effective resolution estimate of 74.7 nm. Comparison of epifluorescence images and the reconstructed super-resolution image of Dendra2 (**D**), mEOS3.2 (**E**) and Dronpa (**F**) fused to tpSil3 with z-focus on the valve region of the diatom. Enlarged details of the fultoportulae to the right. An SEM image in the same scaling that corresponds to the enlarged detail of the fluorescence image is displayed for comparison. Scale bars are 1 μm, and 100 nm for the zoomed images.

After identifying suitable FPs for biosilica embedded super-resolution imaging, we moved forward to perform PALM imaging using Dendra2, mEOS3.2 and Dronpa fused to tpSil3 (SI Movie S1). The high chloroplast background fluorescence impaired single-molecule detection *in vivo*, and thus high resolution imaging (see SI Fig. S1). Additionally, the chloroplasts changed their autofluorescence during 405 nm illumination, which further challenged the identification of single photo-converted molecules (Fig. S3). Therefore, the chloroplasts were removed by extraction of the cells with a detergent-based buffer yielding pure biosilica. The tpSil3 fusion proteins in diatom silica were imaged using a low conversion power and a high imaging power to localize individual Dendra2, mEOS3.2 and Dronpa molecules. After a relatively short recording time of approximately two minutes (1000 frames at 10 frames per second), the movie was analyzed using the super-resolution software ThunderSTORM^37^ and the resulting localizations visualized using a custom-written MATLAB code. Figure 3A shows a reconstructed single-molecule localization image of Dendra2 with the *T. pseudonana* biosilica in valve view and the focal plane in the girdle band region (see Fig. 1B right). By performing multiple line scans perpendicular to the biosilica cylinder, we determined an average full width at half maximum (FWHM) of 76.0 nm ± 4.6 nm (±S.D.). One of those line scans is depicted in Fig. 3B. Taking into account the localization precision per fluorophore of 28 nm (this would be a FWHM of 66 nm assuming a Gaussian distribution) and a linker length of 3 nm (the distance between the chromophore of the FP and tpSil3), the 76 nm can be deconvolved to an underlying thickness of 53 nm ± 3 nm (see SI Fig. S8 for details). This value is in very good agreement with the average thickness of the valve of 60 nm determined by scanning electron microscopy^38^. In comparison, the line scans of the epifluorescence image, which is limited in its resolution by the light diffraction limit, yielded a FWHM of 510 nm ± 55 nm (Fig. 3A,B).

The intricate biosilica pattern of diatoms is most planar in the valve region and can thus be best observed there. We used TIRF imaging to determine the locations of tpSil3-Dendra2 and tpSil3–mEOS3.2 embedded in the biosilica pattern. In epifluorescence images (Fig. 3D,E left), a continuous fluorescence distribution with inhomogeneous intensities over the valve region was observed. In the PALM image (Fig. 3D,E right and Fig. S5), clear fluorescent and non-fluorescent regions can be identified. Interestingly, the large circular non-fluorescent gaps correlate in number and position with the structural elements of fultoportulae observed in wildtype in SEM images (see Fig. 1A and SEM images in Fig. 3D,E). This suggests that tpSil3-Dendra2 and tpSil3-mEOS3.2 are not present within the external tubes of the fultoportulae which are approximately 100 nm in diameter, but rather correlate to the outer region of the fultoportulae basal chamber–a thicker and larger structure (SI Fig. S4). Furthermore, we could not observe a distinct ridge-like pattern in the reconstructed super-resolution images, but a rather sparse localization on the central area of the valve, except for a high density around a central pore like structure. The position and size of this pore-like structure correlates well with the central fultoportulae observed in electron micrographs. We hypothesize that tpSil3 is a major component in the basal chamber of the fultoportulae and plays only a minor role in ridge formation.

We further localized the tpSil3-Dronpa fusion protein, although with a lower localization count per frame (5 versus 14 for mEOS3.2 and Dendra2, SI Fig. S6). This leads to a longer acquisition time (2500 frames) until a similar super-resolution image is formed (Fig. 3F). The lower localization count might originate from a lower expression or bleaching during acquisition due to the UV activation laser.

In the valve regions, 17763 fluorophores (tpSil3-Dendra2) and 14327 fluorophores (tpSil3- mEOS3.2) were localized to form the corresponding super-resolution images. Dendra2 and mEOS3.2 could be localized with an average localization precision^39^ of 28 nm and 25 nm, respectively (SI Fig. S6A). This is in good agreement with previously reported localization precisions for these fluorophores in different biological environments^14^,^40^. Although there have been reports of light trapping^41^ and lensing^42^ effects of the nanostructured diatom frustules we did not experience a pronounced distortion of the far-field image leading to inferior fluorophore localization which indicates that silica-embedding does not affect imaging resolution with these fluorophores. The high amount of localized fluorophores (with a mean of 14 localizations per frame, see SI Fig. S6B) and the localization precision support our hypothesis that the non-fluorescent areas are indeed true gaps in the tpSil3 protein arrangement rather than an artifact generated by sparse image reconstruction. Further analysis of the nearest neighbor distribution clearly peaks under 20 nm for all SMLM images, thus indicating a dense protein pattern (SI Fig. S6C) that satisfies the Nyquist Criterion^43^. Additionally, a Fourier ring correlation was calculated to estimate the resolution^44^. For the super-resolution image in Fig. 3A we achieve according to the Fourier ring correlation criteria a resolution of 74.7 nm (Fig. 3C), which corroborates the observed biosilica FWHM of 76 nm.

## Conclusions

Currently, the high background of chloroplasts hinders *in vivo* single-molecule localization microscopy, however recently developed super-resolution techniques based on fluctuation analysis^45^ are promising approaches for a suppression of the chloroplast background. Despite this limitation, with extracted cells, we achieved localization precisions of 28 nm, 25 nm and 25 nm for silica-embedded tpSil3-Dendra2, tpSil3-mEOS3.2 and tpSil3-Dronpa, respectively. This resolution is well beyond the diffraction limit and allows the investigation of the intricate biosilica pattern observed in EM and if said pattern originates from an underlying protein template for silica biogenesis. Recently reported approaches of correlative super-resolution and electron microscopy might allow additional correlation within a single cell^46^,^47^. Moreover, newly developed fluorescent proteins compatible with a biosilica embedded photo-conversion mechanism may further increase the resolution and thus allow more insights in the underlying protein pattern. Structured illumination microscopy studies showed that biarsenic Cyanine fluorophores (AsCy3 and AsCy3e) can be further used for silica embedded fusion proteins and are due to their blinking nature also potentially suited for single-molecule localization microscopy^13^,^48^. We further anticipate that the use of multicolor PALM and colocalization studies^49^ will allow deeper insights in protein-protein interactions during silica biogenesis. Such insight should be instrumental for understanding the molecular mechanisms that control morphogenesis of the species-specific diatom silica structures with precise nanoscale control.

## Methods

### Cell Cultivation

*Thalassiosira pseudonana* (Hustedt) Hasle et Heimdal clone CCMP1335 was cultured in artificial seawater medium according to the North East Pacific Culture Collection protocol at 18 °C under constant light at 5,000–10,000 lux as previously described^50^.

### Construction of *T. pseudonana* expression vectors

DNA sequences of the photo-activatible FPs with their codons optimized for expression in *T. pseudonana*^51^ were synthesized by GeneArt with restriction sites added to allow for single step cloning into the diatom expression vectors (see SI Table S2). For cytosolic expression of the FPs the plasmid DNA was digested with EcoRV and NotI and ligated into the EcoRV and NotI sites of pTpfcp^30^. For expression of the FPs as a C-terminal gene fusion with tpSil3 the plasmid DNA was digested with EcoRV and NotI and ligated into the EcoRV and NotI sites of pTpfcp/Sil3_nt_^31^. See SI Fig. S7 for a vector scheme including the Sil3-fusion construct.

### Genetic transformation of *T. pseudonana*

The resulting gene constructs were introduced into *T. pseudonana* cells using the Biorad PDS-1000/He particle delivery system as described previously^30^. Co-transformations were performed with the pTpfcp/nat plasmid DNA^30^ for selection of transformed cell lines on agar plates containing 150 μg/mL ClonNat (Jena Biosciences).

### Biosilica isolation

Due to the intense chloroplast autofluorescence for PALM imaging of FPs fused to tpSil3 it was necessary to remove cytosolic components via SDS and EDTA treatment as described^7^. Cell walls were resuspended in PBS prior to imaging.

### Imaging by fluorescence microscopy

Diatoms were imaged on a Nikon N-STORM inverted microscope with a 100x Oil Immersion TIRF objective with a numerical aperture of 1.49. Four fiber-coupled lasers are available for illumination at 405, 488, 561 and 647 nm, respectively. Corresponding filter sets from Semrock, Chroma and AHF were used to block out all unwanted fluorescence and scattered laser light (see SI Mat/Met for a detailed description). The image was projected onto an Andor Ixon 897 EMCCD camera. To visualize the chloroplasts, the red 647 nm laser was used at low power (1.5 mW end of fiber) for 100 ms. For screening of the FPs, the appropriate excitation wavelength (SI Table S1) was chosen at a power of 40 mW and an exposure time of 100 ms. The photo-conversion test was performed by illuminating the sample with 405 nm light for 1 second at 20 mW. Super-resolution images were recorded at 20 fps with an excitation power of 80 mW. Image stacks of at least 1000 images were used (1000 for Fig. 3A,D and SI Fig. S5, 1200 for Fig. 3E, 2500 for Fig. 3F) to ensure a sufficient localization count. For mEOS3.2 and Dendra2 green excitation is sufficient to localize pre-converted FPs. Dronpa is dark in its ground state, a continuous 405 nm activation of 8 mW in addition to the green excitation was required.

### Super-resolution reconstruction

For super-resolution image reconstruction, the software thunderSTORM^37^ was used (see SI for detailed parameter); one of the top performer in a quantitative software evaluation^52^. For visualization, a custom written MATLAB script with a GUI was developed to display super-resolution data and confirm the correct reconstruction. The latter was performed by coloring the individual localization events according to the localization parameter of choice (like localization precision, goodness of fit, etc.). This allows easy identification of outliers and reconstruction quality. See SI Fig. S9 for a GUI screenshot. Scripts and GUI are available upon request.

## Additional Information

**How to cite this article**: Gröger, P. *et al.* Establishing super-resolution imaging for proteins in diatom biosilica. *Sci. Rep.* **6**, 36824; doi: 10.1038/srep36824 (2016).

**Publisher’s note:** Springer Nature remains neutral with regard to jurisdictional claims in published maps and institutional affiliations.

## Supplementary Material

Supplementary Information

Supplementary Movie S1

## Figures and Tables

**Table 1:** Photo-conversion behavior of FPs in diatoms.

FP	Photo-conversion mechanism	Cytosolic expression		Silica embedded		Suitable for silica embedded imaging
		Before activation	After activation	Before activation	After activation	
PATagRFP	PA	Dark	Orange	Dark	Dark	No
PAmCherry1	PA	Dark	Orange	Dark	Dark	No
PA-GFP	PA	Dark	Green	Dark	Dark	No
mEOS3.2	PC	Green	Orange	Green	Orange	Yes
Dendra2	PC	Green	Orange	Green	Orange	Yes
Dronpa	PS	Dark	Green	Dark	Green	Yes

All FPs used for SMLM in diatoms together with the respective screening results are listed. Abbreviations for the conversion mechanism are: photo-activatable (PA), photo-convertible (PC) or photo-switchable (PS). Color names represent the excitation wavelengths 488 nm (green) and 561 nm (orange).
