# Supplementary material for: Establishing super-resolution imaging for proteins in diatom biosilica
Source: Sci Rep. 2016 Nov 9;6:36824. doi: 10.1038/srep36824 (PMC5101815; doi:10.1038/srep36824)
Supplement: Supplementary Information [file srep36824-s1.pdf]

Supplementary Information for

Establishing super-resolution imaging for proteins in diatom biosilica

Philip Gröger[1], Nicole Poulsen[1], Jennifer Klemm[1], Nils Kröger[1,2], Michael Schlierf[1]*

[1]B CUBE – Center for Molecular Bioengineering, TU Dresden, Arnoldstr. 18, 01307 Dresden, Germany
[2] Department of Chemistry and Food Chemistry, TU Dresden, Arnoldstr. 18, 01307 Dresden, Germany

*correspondence: schlierf@bcube-dresden.de

Index

	SI Methods	3
Table S1.	Fluorescent proteins used and their core parameter	4
Figure S1.	Emission spectra of live diatom	5
Figure S2.	Screening of all fluorescent proteins and their activation capability	6
Figure S3.	Photo-conversion analysis of active tpSil3 clones	7
Figure S4.	SEM micrograph of the inside of the valve biosilica	8
Figure S5.	Additional PALM images on tpSil3–Dendra2 and tpSil3-mEOS3.2	9
Figure S6.	Localization parameter for tpSil3-Dendra2, mEOS3.2 and Dronpa	10
Figure S7.	Vector design for cloning	11
Figure S8.	Convolution to estimate tpSil3-Dendra2 filament thickness	12
Figure S9.	MATLAB GUI to visualize super-resolution data from thunderSTORM	13
Table S2.	Codon optimized sequences for the fluorescent proteins	14
Movie S1.	Raw movie of tpSil3-Dendra2, -mEOS3.2 and Dronpa	15

SI Methods

SEM imaging of Thalassiosira pseudonana

Diatoms were lysed (as described under *Biosilica Isolation* in the main methods), dehydrated with ethanol and afterwards critically point dried in a Leica-CPD 300. The dry diatoms were spread onto a carbon conductive adhesive tape and sputter coated with platinum in a Baltec SCD 050. Imaging was performed using a JEOL JSM-7500F scanning electron microscope with a SE2 detector and an acceleration voltage of 15kV.

ThunderSTORM settings

Image filtering was performed using the "à trous" undecimated wavelet transform with a B-spline order of 3 and scale of 2. For localization approximation a local threshold (in an 8-connected neighborhood) of 1x the standard deviation of the filtered image was chosen. The sub-pixel localization was performed via maximum likelihood fitting of the integrated form of a symmetric 2D Gaussian function with a radius of 3 pixel. The initial sigma was 1.6 pixel.

Super-resolution image reconstruction

The visualization of the localized molecules was performed by drawing a normalized symmetric 2D Gaussian for each of them and summing up all the Gaussians to form a final image. As the standard deviation, the localization precision (calculated via the Thompson-Larson-Webb formula as mentioned in the supplementary note for ThunderSTORM) was chosen.

Filter Sets used for imaging

Excitation WL	Laser bandpass	Dichroic	Emission bandpass
405	390/40 BrightLine HC (Semrock)	H 405 LPXR superflat (AHF)	460/50 ET (Chroma)
488	475/35 BrightLine HC (Semrock)	H 488 LPXR superflat (AHF)	525/45 BrightLine HC (Semrock)
561	555/25 ET (Chroma)	H 560 LPXR superflat (AHF)	609/54 BrightLine HC (Semrock)
647	628/40 BrightLine HC (Semrock)	H 643 LPXR superflat (AHF)	700/75 ET (Chroma)

Table S1. Fluorescent proteins used and their core parameter

Abbreviations: λ_{ex} & λ_{em} … excitation and emission spectrum peak position, t_{mature} … maturation time at 37°C, Brightness refers to the product of the extinction coefficient (in Mol^{-1} cm^{-1}) and the quantum yield divided by 1000. The first three FPs are photo-activatable FPs, meaning their non-fluorescent ground state can be activated, irreversibly. MEOS3.2 and Dendra2 are photo-convertible FPs and can be irreversibly switched from green to orange fluorescence. Dronpa is a photo-switchable FPs and can be reversibly switched between a fluorescent and non-fluorescent state.

FP	Transitions	State	λ_{ex} (nm)	λ_{em} (nm)	Brightness	t_{mature} (min)
PATagRFP	Off → Orange, 405 nm	Orange	562	595	25.1	75
PAmCherry1	Off → Orange, 405 nm	Orange	564	595	9.3	23
PA-GFP	Off → Green, 400 nm	Green	504	517	13.7	27
mEOS3.2	Green → Orange, 405 nm	Green<br>Orange	507<br>572	517<br>593	53.2<br>17.7	20
Dendra2	Green → Orange, 480 nm	Green<br>Orange	490<br>553	507<br>573	22.5<br>19.3	90
Dronpa	Off → Green, 400 nm<br>Green → Off, 503 nm	On	503	518	80.7	40

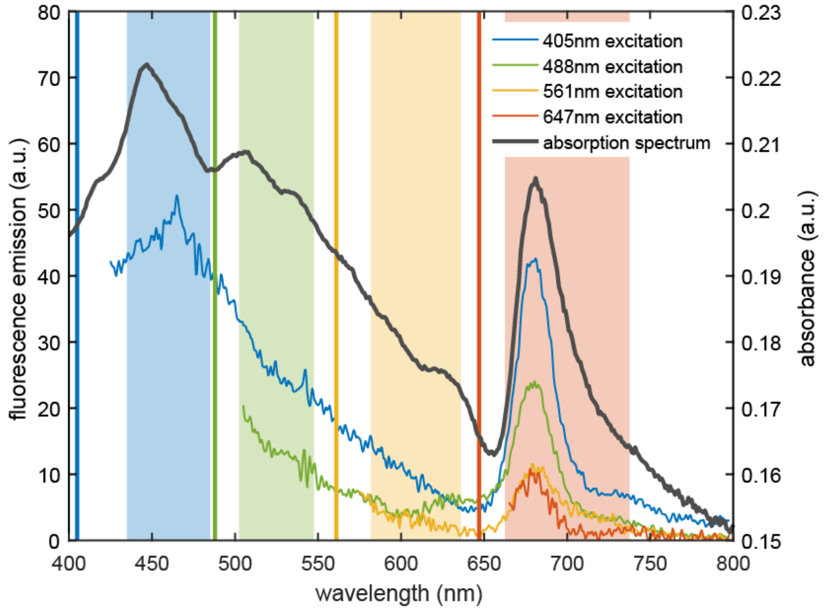

Figure S1. Emission spectra of live diatom

Emission spectra of *T. pseudonana* for different excitation wavelengths. Vertical lines represent the laser lines. The shaded area depict the detection window. The absorption spectrum is plotted in black on a separate y-axis. Clearly visible are the absorption peaks for chlorophyll a at around 670nm and chlorophyll c and fucoxanthin around 450nm.

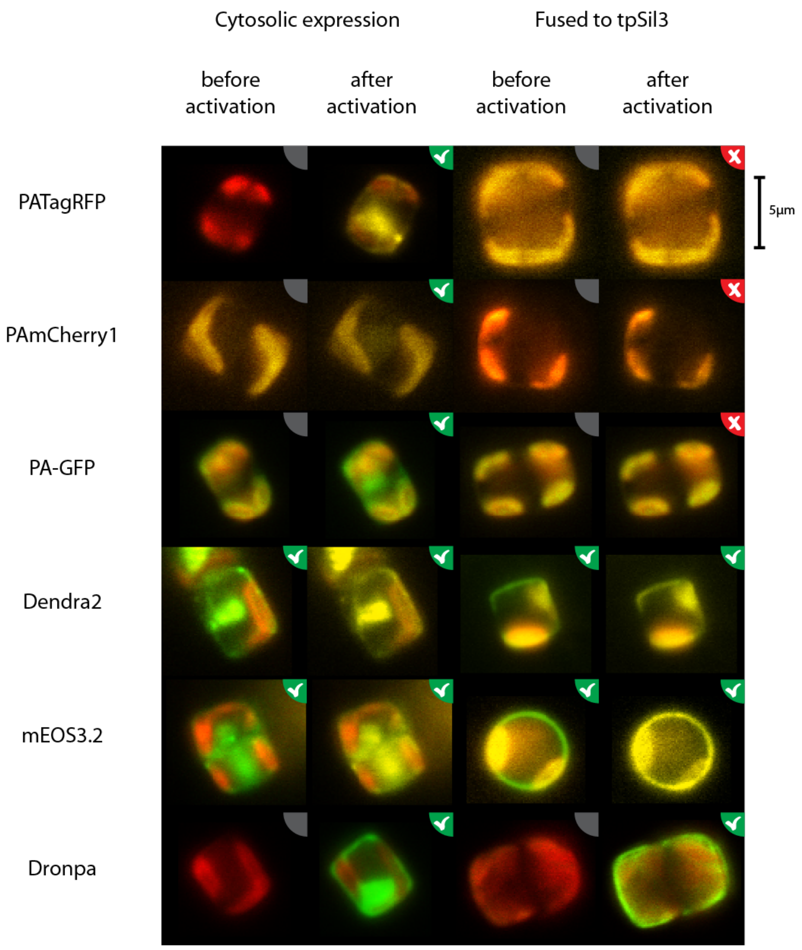

Figure S2. Screening of all fluorescent proteins and their activation capability
Each row displays the activation of a different fluorescent protein. The activation was performed with the appropriate laser light as specified in Table S1. The colors represent the four different excitation wavelengths 405nm (blue), 488nm (green), 561nm (yellow) and 647nm (red). Chloroplasts are always visible in the red channel.

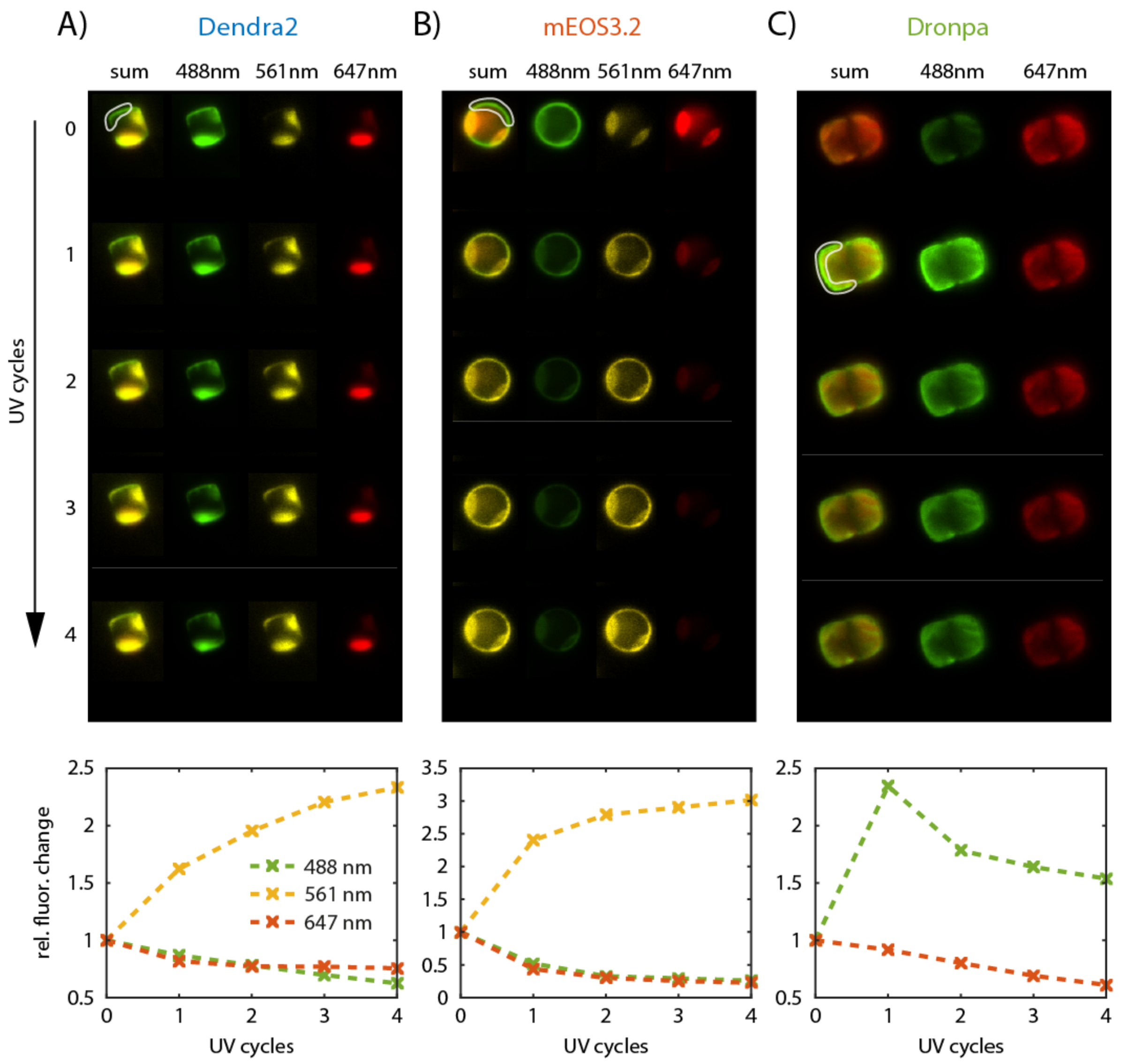

Figure S3. Photo-conversion analysis of active tpSil3 clones

Photo-conversion statistics for Dendra2 (**A**), mEOS3.2 (**B**) and Dronpa (**C**). The top panel show the individual imaging channels during conversion. Each UV cycle equals one second of 405nm illumination at 20mW. To analyze the conversion quantitatively, a region containing the biosilica cell wall and overlapping as little as possible with chloroplasts was selected (depicted in white). The intensity in this region was summed up and the relative intensity change with 488 nm, 561 nm and 647 nm excitation is plotted in the lower panels, showing the clear photo-conversion or –activation of the probes.

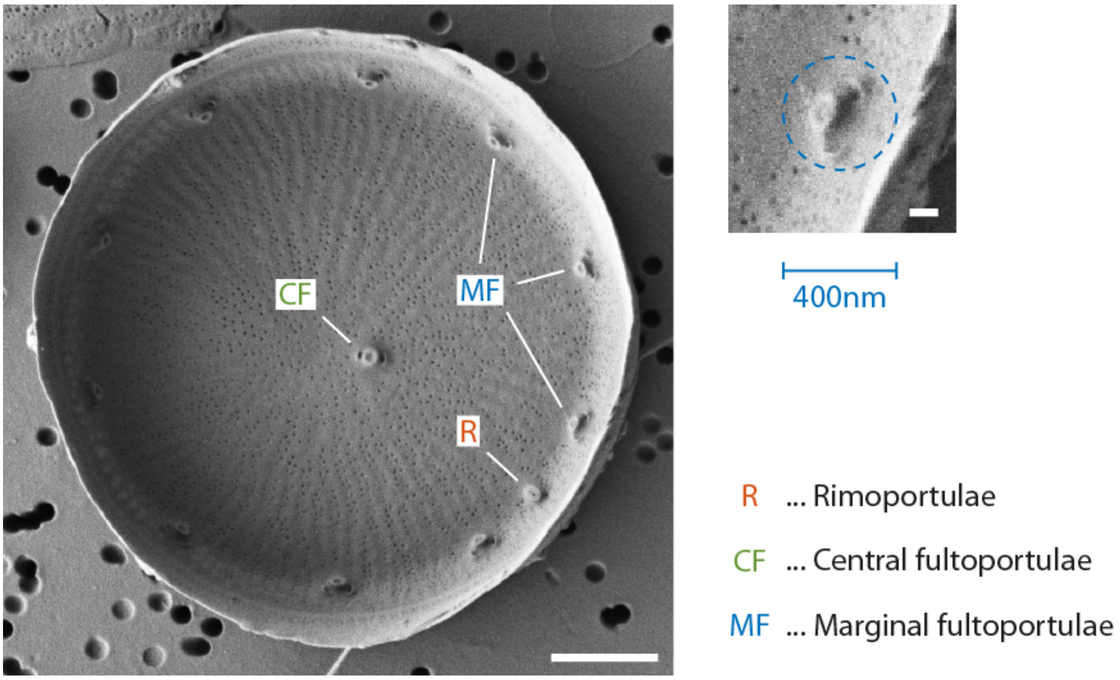

Figure S4. SEM micrograph of the inside of the valve biosilica

SEM image of isolated biosilica valve of *T. pseudonana*. The inside of the valve shows the thick basal chamber of the fultoportulae. A zoomed image shows a diameter estimate of the outer region of the fultoportulae basal chamber of around 400 nm. Scale bars are 1 µm and 100 nm for the zoomed image.

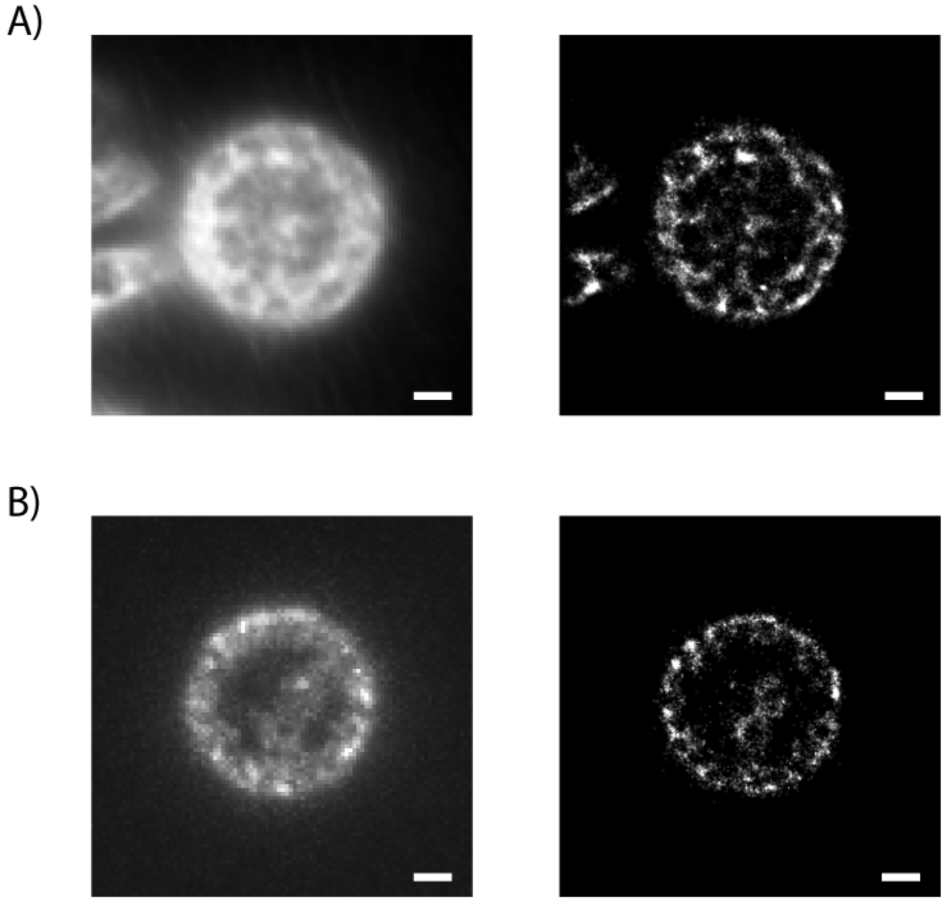

Figure S5. Additional PALM images on tpSil3-Dendra2 and tpSil3-mEOS3.2
Comparison of epifluorescence images (left) and the reconstructed super-resolution image (right) of (**A**) tpSil3-Dendra2 and (**B**) tpSil3-mEOS3.2 with z-focus on the valve region of the diatom. All scale bars are 1μm.

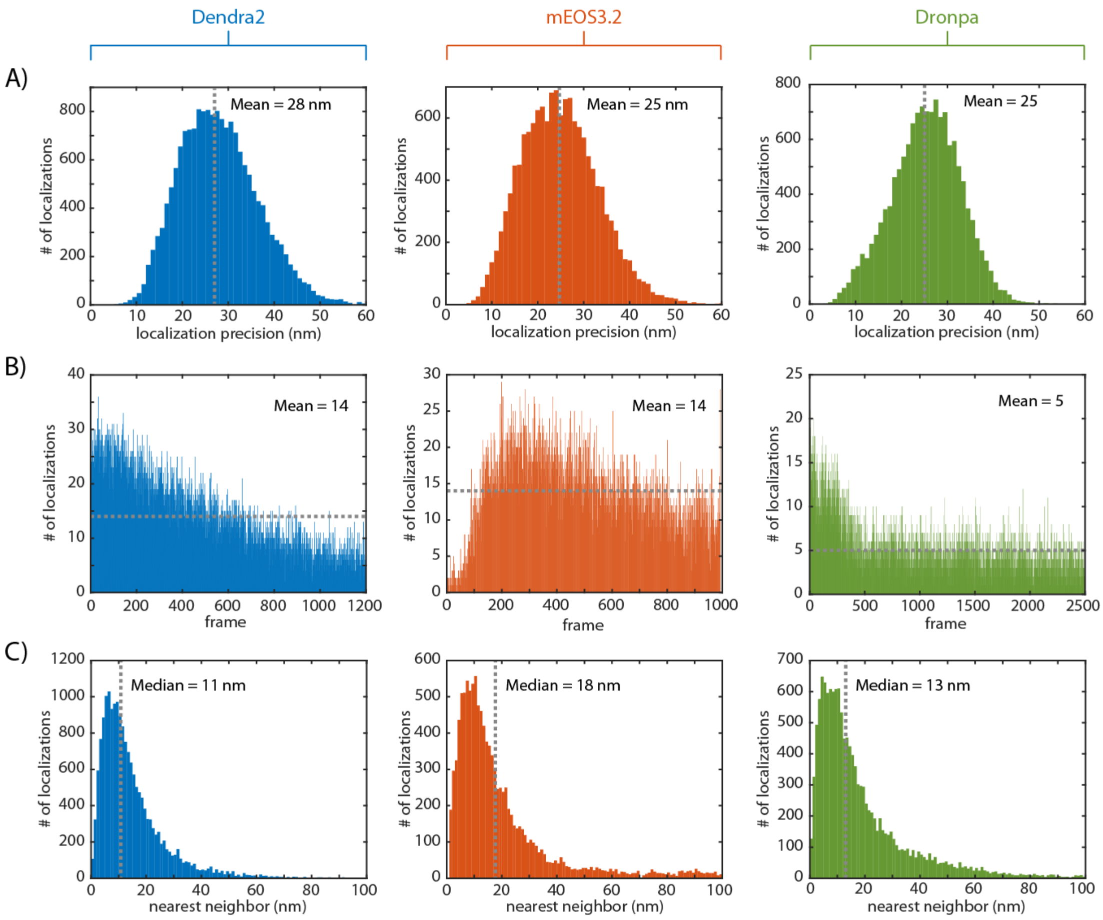

Figure S6. Localization parameter for tpSil3-Dendra2, mEOS3.2 and Dronpa
Parameter of the reconstructed super-resolution image in Figure 2D, E and F for Dendra2, mEOS3.2 and Dronpa, respectively. (**A**) Localization precision of all localizations. (**B**) Number of localized events per frame. (**C**) Nearest neighbor distribution of all localized events.

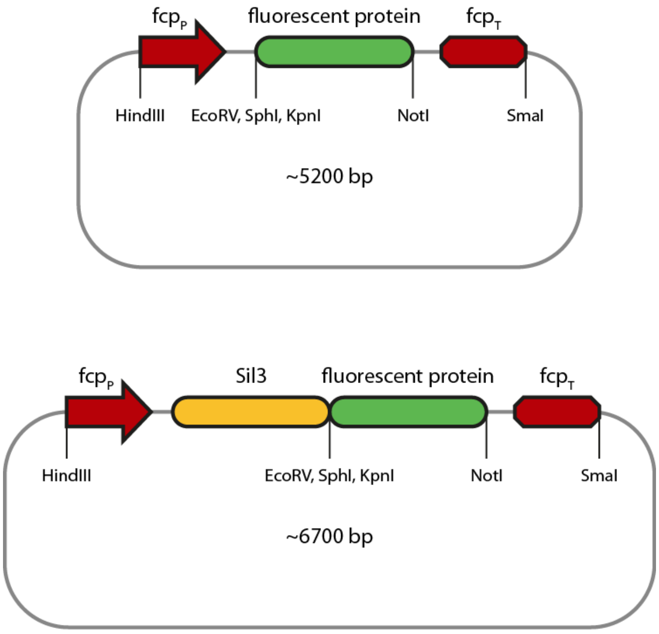

Figure S7. Vector design for cloning

(**Top**) Cytosolic expression featuring the fluorescent protein in between the fcp promoter and terminator.
(**Bottom**) Sil3 fusion constructs, with an N-terminal Sillafin 3 followed by the C-terminal FPs

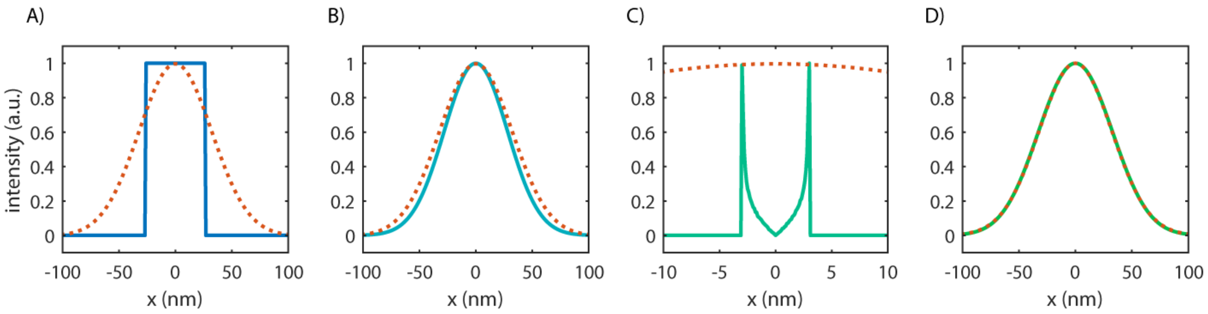

Figure S8. Convolution to estimate tpSil3-Dendra2 filament thickness

Convolution process to estimate the underlying filament thickness based on a measured filament thickness of 76nm (FWHM) in the super-resolution image. The gauss profile of the measured filament is displayed in red in all plots. (**A**) Underlying filament with a thickness of 53nm. (**B**) The first convolution contribution: Fluorophore localization uncertainty of 28nm. (**C**) The second convolution contribution: Linker length between protein and fluorophore of 3nm. Assumed as points on a sphere of 3nm radius. (**D**) Final convolution of A) to C) resulting in the desired width of 76nm.

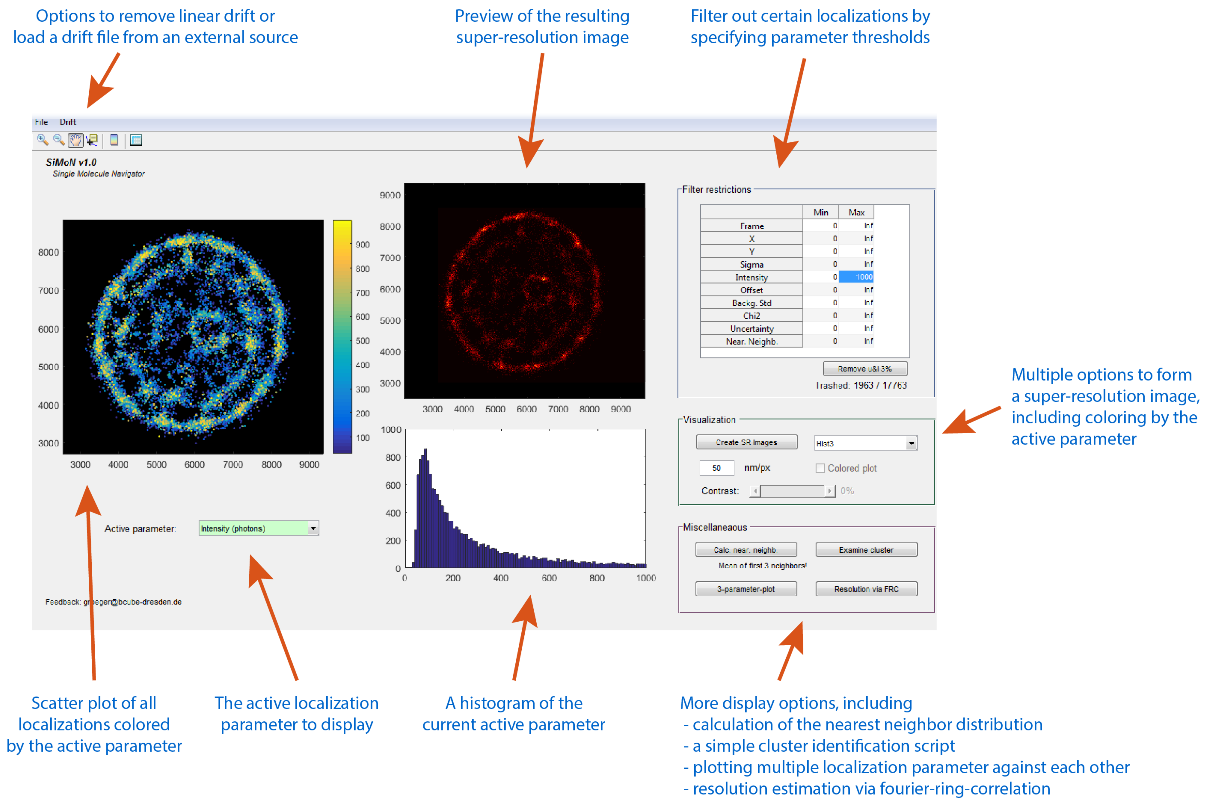

Figure S9. MATLAB GUI to visualize super-resolution data from thunderSTORM
Screenshot of the custom written MATLAB GUI for super-resolution data visualization with several features annotated.

Table S2. Codon optimized sequences for the fluorescent proteins

DNA sequences of all the fluorescent proteins used in this study - plus their translation. Codon optimized for expression in *T. pseudonana*.

PATagRFP

702 nucleotides, 234 amino acids

```
  1 ATGTCCGAGTTGATCAAAGAAAACATGCAC ATGAAGTTGTACATGGAAGGTACTGTGAAC AACCACCACTTCAAGTGCACATCAGAGGGC GAGGGAAAGCCATACGAGGGAACACAAACA ATGCGTATCAAGGTGGTGGAAGGTGGACCA
  1  M  S  E  L  I  K  E  N  M  H   M  K  L  Y  M  E  G  T  V  N   N  H  H  F  K  C  T  S  E  G   E  G  K  P  Y  E  G  T  Q  T   M  R  I  K  V  V  E  G  G  P
151 TTGCCATTCGCATTCGACATCTTGGCCACG AGCTTTATGTACGGATCCTCCACATTCATC AACCACACGCAGGGAATCCCAGACTTCTGG AAGCAATCATTCCCAGAGGGATTCACATGG GAGCGTGTGACAACATACGAGGATGGTGGT
 51  L  P  F  A  F  D  I  L  A  T   S  F  M  Y  G  S  S  T  F  I   N  H  T  Q  G  I  P  D  F  W   K  Q  S  F  P  E  G  F  T  W   E  R  V  T  T  Y  E  D  G  G
301 GTTTTGACGGCAACGCAAGACACCTCATTG CAAGACGGATGCTTGATCTACAACGTGAAG ATCAGGGGAGTGAACTTCCCATCAAACGGA CCAGTGATGAAGAAAAAGACGTTGGGATGG GAGCCATCCACCGAGAAGTTGAAGCCAGCA
101  V  L  T  A  T  Q  D  T  S  L   Q  D  G  C  L  I  Y  N  V  K   I  R  G  V  N  F  P  S  N  G   P  V  M  K  K  K  T  L  G  W   E  P  S  T  E  K  L  K  P  A
451 GATGGTGGATTGGAGGGAAGAGTGGATATG GCATTGAAGTTGGTTGGAGGTGGACACTTG ATCTGCAACTTCAAGACGACGTACCGTTCC AAGAAGCCAGCCAAGAACTTGAAGATGCCT GGTGTTTACTACGTGGACCGTCGTTTGGAG
151  D  G  G  L  E  G  R  V  D  M   A  L  K  L  V  G  G  G  H  L   I  C  N  F  K  T  T  Y  R  S   K  K  P  A  K  N  L  K  M  P   G  V  Y  Y  V  D  R  R  L  E
601 ATCATCAAAGAGGCCGACAAAGAGACATAC TGGGAGCAACATGAGGTGGCAGTGGCAAGA TACTCCGACTTGCCATCAAAGTTGGGACAC AAGTTGAACTGA
201  I  I  K  E  A  D  K  E  T  Y   W  E  Q  H  E  V  A  V  A  R   Y  S  D  L  P  S  K  L  G  H   K  L  N  *
```

PAmCherry

711 nucleotides, 237 amino acids

```
  1 ATGGTGTCAAAGGGTGAAGAGGACAACATG GCCATCATCAAAGAATTCATGCGTTTCAAG GTGCACATGGAAGGATCCGTGAACGGACAT GTGTTTGAGATTGAGGGTGAAGGCGAAGGA CGACCATACGAGGGAACACAGACAGCAAAG
  1  M  V  S  K  G  E  E  D  N  M   A  I  I  K  E  F  M  R  F  K   V  H  M  E  G  S  V  N  G  H   V  F  E  I  E  G  E  G  E  G   R  P  Y  E  G  T  Q  T  A  K
151 TTGAAAGTGACGAAGGGTGGACCATTGCCA TTCACGTGGGATATTTTGTCGCCACAGTTT ATGTACGGATCCAACGCATACGTGAAGCAC CCAGCAGATATTCCAGACTACTTCAAGTTG TCCTTCCCAGAGGGATTCAAGTGGGAGCGT
 51  L  K  V  T  K  G  G  P  L  P   F  T  W  D  I  L  S  P  Q  F   M  Y  G  S  N  A  Y  V  K  H   P  A  D  I  P  D  Y  F  K  L   S  F  P  E  G  F  K  W  E  R
301 GTGATGAAGTTTGAGGATGGTGGTGTTGTG ACCGTGACGCAGGATTCATCATTGCAAGAC GGTGAATTCATCTACAAAGTGAAGTTGCGT GGAACGAACTTCCCATCCGATGGACCAGTG ATGCAGAAAAAGACAATGGGATGGGAGGCC
101  V  M  K  F  E  D  G  G  V  V   T  V  T  Q  D  S  S  L  Q  D   G  E  F  I  Y  K  V  K  L  R   G  T  N  F  P  S  D  G  P  V   M  Q  K  K  T  M  G  W  E  A
451 TTGTCAGAGAGAATGTACCCAGAGGATGGT GCCTTGAAGGGTGAAGTTAAGCCACGTGTG AAGTTGAAAGACGGTGGACACTACGATGCA GAAGTCAAGACGACGTACAAGGCAAAGAAG CCAGTTCAGTTGCCTGGTGCATACAACGTG
151  L  S  E  R  M  Y  P  E  D  G   A  L  K  G  E  V  K  P  R  V   K  L  K  D  G  G  H  Y  D  A   E  V  K  T  T  Y  K  A  K  K   P  V  Q  L  P  G  A  Y  N  V
601 AACCGAAAGTTGGACATCACGTCCCACAAC GAGGATTACACGATCGTTGAGCAATACGAG AGAGCAGAGGGACGTCATAGTACAGGTGGA ATGGACGAGTTGTACAAATGA
201  N  R  K  L  D  I  T  S  H  N   E  D  Y  T  I  V  E  Q  Y  E   R  A  E  G  R  H  S  T  G  G   M  D  E  L  Y  K  *
```

PAGFP

720 nucleotides, 240 amino acids

```
  1 ATGGTGAGCAAGGGCGAGGAGCTGTTCACC GGGGTGGTGCCCATCCTGGTCGAGCTGGAC GGCGACGTAAACGGCCACAAGTTCAGCGTG TCCGGCGAGGGCGAGGGCGATGCCACCTAC GGCAAGCTGACCCTGAAGTTCATCTGCACC
  1  M  V  S  K  G  E  E  L  F  T   G  V  V  P  I  L  V  E  L  D   G  D  V  N  G  H  K  F  S  V   S  G  E  G  E  G  D  A  T  Y   G  K  L  T  L  K  F  I  C  T
151 ACCGGCAAGCTGCCCGTGCCCTGGCCCACC CTCGTGACCACCTTCAGCTACGGCGTGCAG TGCTTCAGCCGCTACCCCGACCACATGAAG CAGCACGACTTCTTCAAGTCCGCCATGCCC GAAGGCTACGTCCAGGAGCGCACCATCTTC
 51  T  G  K  L  P  V  P  W  P  T   L  V  T  T  F  S  Y  G  V  Q   C  F  S  R  Y  P  D  H  M  K   Q  H  D  F  F  K  S  A  M  P   E  G  Y  V  Q  E  R  T  I  F
301 TTCAAGGACGACGGCAACTACAAGACCCGC GCCGAGGTGAAGTTCGAGGGCGACACCCTG GTGAACCGCATCGAGCTGAAGGGCATCGAC TTCAAGGAGGACGGCAACATCCTGGGGCAC AAGCTGGAGTACAACTACAACAGCCACAAC
101  F  K  D  D  G  N  Y  K  T  R   A  E  V  K  F  E  G  D  T  L   V  N  R  I  E  L  K  G  I  D   F  K  E  D  G  N  I  L  G  H   K  L  E  Y  N  Y  N  S  H  N
451 GTCTATATCATGGCCGACAAGCAGAAGAAC GGCATCAAGGCCAACTTCAAGATCCGCCAC AACATCGAGGACGGCAGCGTGCAGCTCGCC GACCACTACCAGCAGAACACCCCCATCGGC GACGGCCCCGTGCTGCTGCCCGACAACCAC
151  V  Y  I  M  A  D  K  Q  K  N   G  I  K  A  N  F  K  I  R  H   N  I  E  D  G  S  V  Q  L  A   D  H  Y  Q  Q  N  T  P  I  G   D  G  P  V  L  L  P  D  N  H
601 TACCTGAGCCACCAGTCCAAGCTGAGCAAA GACCCCAACGAGAAGCGCGATCACATGGTC CTGCTGGAGTTCGTGACCGCCGCCGGGATC ACTCTCGGCATGGACGAGCTGTACAAGTAA
201  Y  L  S  H  Q  S  K  L  S  K   D  P  N  E  K  R  D  H  M  V   L  L  E  F  V  T  A  A  G  I   T  L  G  M  D  E  L  Y  K  *
```

mEOS3.2

681 nucleotides, 227 amino acids

```
  1 ATGTCTGCAATCAAGCCAGACATGAAGATC AAGTTGCGTATGGAAGGCAACGTGAACGGA CACCATTTCGTGATCGACGGTGATGGAACT GGAAAGCCATTCGAGGGAAAGCAGTCCATG GATTTGGAAGTGAAAGAGGGTGGACCATTG
  1  M  S  A  I  K  P  D  M  K  I   K  L  R  M  E  G  N  V  N  G   H  H  F  V  I  D  G  D  G  T   G  K  P  F  E  G  K  Q  S  M   D  L  E  V  K  E  G  G  P  L
151 CCATTCGCATTCGACATCTTGACAACGGCC TTCCATTACGGAAACCGTGTGTTCGCAAAG TACCCAGACAACATCCAGGACTACTTCAAG CAGTCCTTCCCAAAGGGATACTCCTGGGAG AGATCATTGACATTCGAGGACGGTGGAATC
 51  P  F  A  F  D  I  L  T  T  A   F  H  Y  G  N  R  V  F  A  K   Y  P  D  N  I  Q  D  Y  F  K   Q  S  F  P  K  G  Y  S  W  E   R  S  L  T  F  E  D  G  G  I
301 TGCAACGCACGTAACGATATTACGATGGAA GGCGACACGTTCTACAACAAAGTGCGATTC TACGGAACGAACTTCCCAGCAAACGGACCA GTGATGCAGAAAAAGACATTGAAGTGGGAG CCATCCACCGAGAAGATGTACGTGCGTGAT
101  C  N  A  R  N  D  I  T  M  E   G  D  T  F  Y  N  K  V  R  F   Y  G  T  N  F  P  A  N  G  P   V  M  Q  K  K  T  L  K  W  E   P  S  T  E  K  M  Y  V  R  D
451 GGTGTCTTGACGGGTGATATTGAGATGGCC TTGTTGTTGGAGGGAAACGCACATTACCGT TGCGATTTCCGTACGACGTACAAGGCCAAA GAAAAGGGTGTTAAGTTGCCAGGTGCACAC TTCGTGGATCACTGCATCGAGATCTTGTCC
151  G  V  L  T  G  D  I  E  M  A   L  L  L  E  G  N  A  H  Y  R   C  D  F  R  T  T  Y  K  A  K   E  K  G  V  K  L  P  G  A  H   F  V  D  H  C  I  E  I  L  S
601 CACGACAAGGACTATAACAAAGTGAAGTTG TACGAGCACGCAGTGGCACACTCAGGATTG CCAGATAACGCAAGACGTTGA
201  H  D  K  D  Y  N  K  V  K  L   Y  E  H  A  V  A  H  S  G  L   P  D  N  A  R  R  *
```

Dendra2

693 nucleotides, 231 amino acids

```
  1 ATGAACACACCTGGAATCAACTTGATCAAA GAAGATATGCGAGTCAAGGTGCACATGGAA GGCAACGTGAACGGACATGCATTCGTGATT GAGGGCGAGGGAAAGGGAAAACCATACGAG GGAACACAGACGGCCAACTTGACAGTGAAA
  1  M  N  T  P  G  I  N  L  I  K   E  D  M  R  V  K  V  H  M  E   G  N  V  N  G  H  A  F  V  I   E  G  E  G  K  G  K  P  Y  E   G  T  Q  T  A  N  L  T  V  K
151 GAGGGTGCACCATTGCCATTCTCGTACGAC ATTTTGACAACGGCCGTGCATTACGGAAAC CGTGTGTTTACAAAGTACCCAGAGGACATC CCAGACTACTTCAAGCAATCATTCCCAGAG GGATACTCGTGGGAGCGTACAATGACATTC
 51  E  G  A  P  L  P  F  S  Y  D   I  L  T  T  A  V  H  Y  G  N   R  V  F  T  K  Y  P  E  D  I   P  D  Y  F  K  Q  S  F  P  E   G  Y  S  W  E  R  T  M  T  F
301 GAGGATAAGGGAATCTGCACGATCCGTTCC GACATTTCATTGGAGGGCGACTGTTTCTTC CAGAACGTGCGTTTCAAGGGAACGAACTTT CCACCAAACGGACCAGTGATGCAGAAAAAG ACATTGAAGTGGGAGCCATCCACCGAGAAG
101  E  D  K  G  I  C  T  I  R  S   D  I  S  L  E  G  D  C  F  F   Q  N  V  R  F  K  G  T  N  F   P  P  N  G  P  V  M  Q  K  K   T  L  K  W  E  P  S  T  E  K
451 TTGCATGTTCGTGACGGTTTGTTGGTCGGA AACATCAACATGGCCTTGTTGTTGGAGGGT GGTGGACACTACTTGTGCGATTTCAAGACG ACGTACAAGGCCAAGAAGGTGGTCCAATTG CCAGATGCACATTTCGTGGATCACCGTATC
151  L  H  V  R  D  G  L  L  V  G   N  I  N  M  A  L  L  L  E  G   G  G  H  Y  L  C  D  F  K  T   T  Y  K  A  K  K  V  V  Q  L   P  D  A  H  F  V  D  H  R  I
601 GAGATCTTGGGAAACGACTCCGACTACAAC AAAGTGAAGTTGTACGAGCACGCAGTGGCC AGATACTCTCCATTGCCATCACAAGTCTGG TGA
201  E  I  L  G  N  D  S  D  Y  N   K  V  K  L  Y  E  H  A  V  A   R  Y  S  P  L  P  S  Q  V  W   *
```

Dronpa

678 nucleotides, 226 amino acids

```
  1 ATGGTGTCAGTGATTAAGCCAGACATGAAG ATCAAGTTGCGTATGGAAGGTGCAGTGAAC GGACATCCATTCGCAATTGAGGGTGTTGGA TTGGGAAAGCCATTCGAGGGAAAGCAGTCG ATGGACTTGAAAGTGAAAGAGGGTGGACCA
  1  M  V  S  V  I  K  P  D  M  K   I  K  L  R  M  E  G  A  V  N   G  H  P  F  A  I  E  G  V  G   L  G  K  P  F  E  G  K  Q  S   M  D  L  K  V  K  E  G  G  P
151 TTGCCATTCGCCTACGATATTTTGACGACG GTGTTCTGCTACGGAAACCGTGTGTTCGCA AAGTACCCAGAGAACATCGTGGACTACTTC AAGCAGTCATTCCCAGAGGGATACTCGTGG GAGAGATCAATGAACTACGAGGACGGTGGA
 51  L  P  F  A  Y  D  I  L  T  T   V  F  C  Y  G  N  R  V  F  A   K  Y  P  E  N  I  V  D  Y  F   K  Q  S  F  P  E  G  Y  S  W   E  R  S  M  N  Y  E  D  G  G
301 ATCTGCAACGCAACAAACGATATTACGTTG GACGGTGACTGCTACATCTACGAGATCCGT TTCGATGGTGTCAACTTCCCAGCAAACGGA CCAGTGATGCAAAAGCGTACAGTGAAGTGG GAGCCATCCACAGAGAAGTTGTACGTGCGT
101  I  C  N  A  T  N  D  I  T  L   D  G  D  C  Y  I  Y  E  I  R   F  D  G  V  N  F  P  A  N  G   P  V  M  Q  K  R  T  V  K  W   E  P  S  T  E  K  L  Y  V  R
451 GATGGTGTCTTGAAGGGTGACGTGAACATG GCATTGTCATTGGAGGGTGGTGGACACTAC CGTTGCGATTTCAAGACGACGTACAAGGCA AAGAAGGTGGTCCAATTGCCAGACTACCAT TTCGTGGATCACCACATCGAGATCAAGTCC
151  D  G  V  L  K  G  D  V  N  M   A  L  S  L  E  G  G  G  H  Y   R  C  D  F  K  T  T  Y  K  A   K  K  V  V  Q  L  P  D  Y  H   F  V  D  H  H  I  E  I  K  S
601 CACGACAAGGACTACTCCAACGTGAACTTG CATGAGCACGCAGAGGCACATTCAGAGTTG CCACGTCAAGCCAAATGA
201  H  D  K  D  Y  S  N  V  N  L   H  E  H  A  E  A  H  S  E  L   P  R  Q  A  K  *
```

(see attached movie file)

Movie S1. Raw movie of tpSil3-Dendra2, -mEOS3.2 and Dronpa

The movie contains the first 500 frames of the raw movie used to create the PALM images shown in this study (Fig. 2). From left to right: Dendra2, mEOS3.2 and Dronpa. One pixel equals 106.7 nm.
